# Ecological factors influencing parenting self-efficacy among working mothers with a child under 36 month old in South Korea: a cross‐sectional and correlational study

**DOI:** 10.1186/s12905-022-01639-8

**Published:** 2022-03-05

**Authors:** Ju-Eun Song, Eun Ha Roh, Hyun-Ju Chae, Tiffany Kim

**Affiliations:** 1 College of Nursing, Ajou University, Suwon, Republic of Korea; 2 Global Korean Nursing Foundation, Seoul, Republic of Korea; 3 Department of Nursing, Joongbu University, 201, Daehak-ro, Chubu-myeon, Geumsan-gun, Chungnam, 32713 Republic of Korea; 4 School of Nursing, Bouvé College of Health Sciences, Northeastern University, Boston, MA USA

**Keywords:** Mothers, Employment, Parenting, Self-efficacy, Ecology

## Abstract

**Background:**

Parenting self-efficacy is an essential component for parents to successfully perform their role and is important for mother and child well-being. To support parenting self-efficacy amongst working mothers, it is necessary to understand the factors influencing parenting self-efficacy amongst this group. However, the majority of previous studies regarding factors influencing parenting self-efficacy did not focus on working mothers. Therefore, this study aimed to identify the factors influencing parenting self-efficacy of working mothers using an ecological framework.

**Methods:**

The research design was a cross-sectional, correlational study. The participants were 298 working mothers with a child under 3 years of age, who were recruited from ten nurseries. Data were collected from August 8 to September 22, 2017 using structured questionnaires, including the Parenting Sense of Competency scale, a one-item Short Form Health Survey scale, the Maternal Role Satisfaction scale, the Parenting Stress Inventory, the Work and Parent Role Conflict scale, the Parenting Alliance Inventory, the Social Support scale, and the Childbirth and Parenting Friendly System scale. The study process of this study was approved by the Institutional Review Board. Collected data were analyzed by SPSS 23.0 Win program with descriptive statistics, t-test, one way ANOVA, Pearson correlation coefficient, and hierarchical multiple regression.

**Results:**

Working mothers who were the primary caregiver had higher parenting self-efficacy compared to those who were not the primary caregiver (β = .13, *p* = .022). At the individual level, the higher maternal role satisfaction, the higher parenting self-efficacy of working mothers (β = .27, *p* < .001). In the micro-system level, higher parenting support by a spouse was associated with higher parenting self-efficacy of working mothers (β = .19, *p* = .002).

**Conclusions:**

Educational interventions for increasing the awareness and satisfaction of maternal role and various strategies for fathers' active participation in parenting should be developed. In addition, practical interventions that reduce the burden of parenting while supporting parenting self-efficacy of working mothers who are the primary caregiver should also be considered.

## Background

Parenting self-efficacy (PSE) is a multidimensional concept defined as parental beliefs or confidence in their ability to successfully perform the tasks associated with parenting [1–3]. This encompasses both level of knowledge about appropriate child-rearing behaviors and degree of confidence in one's ability to perform parenting tasks [2, 4]. PSE is an essential component for parents to successfully perform their role [3, 4] and a crucial factor in facilitating a smooth transition to parenthood [5, 6].

Becoming a parent is one of the most significant experiences in a women's life, but it can be a stressful time for new mothers [1, 7]. The stress associated with parenting interferes with a mother's transition to motherhood and acts as a risk factor for various health and developmental problems in childhood [8]. In contrast, maternal PSE is associated with a number of positive outcomes for a mother and her children [1, 2] and acts as a protective factor against developmental delay in at-risk family environments [9]. Therefore, improving maternal PSE is critical to promote the well-being of both mother and child.

To improve maternal PSE, a better understanding of the factors influencing maternal PSE is needed [2, 6]. Previous studies reported that multiple factors such as maternal identity, general self-efficacy, parental fatigue, parenting stress, parenting satisfaction, marital satisfaction, social support, children’s temperament, maternal age, number of children, and family income were associated with maternal PSE [10–15]. However, most of these studies were not focused on working mothers. Women's participation in paid work is increasing dramatically worldwide [16, 17]. The responsibilities of parenting are undoubtedly challenging for all mothers [17] and these challenges are likely even greater for working mothers [18]. For example, previous studies have reported low overall maternal PSE scores (31.6 ~ 48.1 out of 68) [6, 15], with the maternal PSE of working mothers even lower than that of non-working mothers [15]. In addition, most of these studies were conducted in a piecemeal fashion or did not use a theoretical framework [13, 14]. Maternal PSE is a highly complex and multi-faced concept [3, 19], so the use of a guiding theory to conceptualize how different variables work together to determine maternal PSE can help increase our understanding [20]. Therefore, in order to support successful parenting and transition to motherhood of working mothers, additional research is needed to identify the factors influencing maternal PSE specifically for working mothers with the application of an appropriate theoretical framework.

Maternal PSE is multi-faced concept and can be influenced by various aspects of the environment under self-efficacy theory [19, 21]. Considering these, Bronfenbrenner’s ecological model [22] can be applied as a theoretical foundation for influencing factors of PSE. The ecological model explains that individual’s development or behaviors are influenced by various environment system surrounding individuals [22, 23] and was used in previous studies related to mother’s adaptation after childbirth [24, 25]. Maternal PSE, a developmental outcome of motherhood, is not only affected by her own characteristics (the individual level) such as satisfactory feelings or stressful experiences related to parenting [8, 12], but also by the nearest environment around her (the micro-system level) including her partner and significant others [11, 13, 15]. Maternal PSE is also influenced by the broader social setting (the exo-system level) such as support systems and polices for parenting [26]. Therefore, we conducted this study to identify the factors influencing PSE of working mothers using various variables nested within the ecological model.

## Methods

### Design

This study was a cross‐sectional and correlational design with a self‐report questionnaire.

### Participants

A convenience sample of 300 participants was recruited from August 8 to September 22, 2017 at ten nurseries which were willing to assist with data collection in Seoul and Gyeonggi province, South Korea. Participants were included if they were 1) working mothers having a child under 3 months to 3 years of age and 2) agreed to participate in the study voluntarily. The exclusion criteria of this study were 1) women not working outside the home or 2) working mothers having a child under 3 month old. The reason to exclude working mothers having a young child under 3 months was that the maternal leave in the South Korea is generally 3 months after childbirth, thus most working mothers do not start their work again until 4 months.

For recruitment of participants, the research team contacted the administrators of nurseries who were willing to participate in the study using the researchers’ human network. After receiving permission for study participation, we explained the inclusion and exclusion criteria of participants to the administrators of the nurseries. The administrator of each nursery screened the mothers for inclusion, and contacted the eligible mothers to request consent for contact from the research team. The research team received the contact details of 300 working mothers from ten nurseries, who met inclusion criteria and had consented to contact. Among the 300 women contacted, 298 participants returned the questionnaires to the research team (response rate of 99.3%). This whole process took one and half months total. Using the G*Power 3.1 software program for a post‐hoc power analysis of regression, the sample size of this study (N = 298) reached a power (1‐β) of 99.9%, with a conventional medium effect size of 0.15, an alpha value of 0.05, and ten independent variables [27].

### Instrument

Data were collected using a self‐report questionnaire that contained information about general characteristics, as well as parenting efficacy and its affecting variables nested in the various levels of environments surrounding mother.

*Parenting self-efficacy* was measured with the Parenting Sense of Competency scale, developed by Gibaud‐Wallston and Wandersman [28], and translated into Korean by Jeong [29]. It consists of nine items using a five point Likert scale (1–5), resulting in scores from nine to 45, with higher scores indicating higher levels of parenting efficacy. The internal consistency reliability using Cronbach's alpha was 0.93 [29], and 0.81 in this study.

*Perceived health status* was the mothers' subjective rating of their own health condition and was measured by a one-item Short Form Health Survey scale with a ten-point numerical rating scale developed by Stewart et al. [30] and translated into Korean by Son et al. [31]. A rating of zero means "I do not feel at all healthy" and 10 means "I feel that I could not be healthier". Scores ranged from 0 to 10, with higher scores indicating higher level of subjective health status. A ten-point single item numerical rating scale is widely used to measure subjective feelings [32] and the validity of this subjective rating scale was reported in a previous study in Korea [31].

*Maternal role satisfaction* was measured by one-item Maternal Role Satisfaction scale, a ten-point numerical rating scale, developed by the research team because it is considered an acceptable measure of subjective feelings [32]. A rating of zero means " I am not satisfied at all as a mother." and 10 means "I am supremely satisfied as a mother". Scores ranged from 0 to 10, with higher scores indicating higher levels of satisfaction as a mother.

*Parenting stress* was measured with the Parenting Stress Inventory, developed by Kim and Kang [33]. It has three substructures: daily life stress of parenting (10 items), parenting role performance stress (12 items), and guilty feeling for parenting by others (8 items). It consists of 32 items using a five point Likert scale (1–5), resulting in scores from 32 to 160, with higher scores indicating higher levels of parenting stress. The internal consistency reliability using Cronbach's alpha was 0.95 in this study.

*Work-parent role conflict* was measured with a Work and Parent Role Conflict scale developed by Seo [34] based on the Work Spillover Scale (WSS) [35]. It consists of 8 items using a five point Likert scale (1–5), resulting in score 8 to 40, with higher scores indicating higher levels of role conflict between work and parenting. The internal consistency reliability using Cronbach's alpha was 0.91 in this study.

*Parenting support by spouse* was measured with the Parenting Alliance Inventory, originally developed by Abidin [36], and translated into Korean by Shin [37]. It has 14 items using a five point Likert scale (1–5), resulting in scores from 14 to 70, with higher scores indicating higher levels of childcare support from husbands. The internal consistency reliability using Cronbach's alpha was 0.85 [37], and 0.92 in this study.

*Social support* was measured with the Social Support scale, developed by Jang et al. [38], which included various types of supports related to physical, psychological, and time sharing for mothers. It consists of 19 items using a four point Likert scale (1–4), resulting in scores from 19 to 76, with higher scores indicating higher levels of social support. The internal consistency reliability using Cronbach's alpha was 0.97 [38], and 0.94 in this study.

*Childbirth and parenting friendly workplace system* is the various workplace policies to support childbirth and parenting in the workplace, and was measured with the Childbirth and Parenting Friendly System scale developed by Choi [39]. It consists of 7 policies to support childbirth and parenting, i.e., maternity leave, parental leave, abortion leave, financial support for childcare, fetal examination leave, breastfeeding room, and feeding time allowance in the workplace. Each item was measured using a dichotomous scale of yes (1) or no (0), resulting in score 0 to 7, with higher score indicating higher levels of supportive workplace environment for childbirth and parenting. The internal consistency reliability using Cronbach's alpha was 0.97 [39], and 0.71 in this study.

### Data collection and ethical consideration

Data were collected from August 8 to September 22, 2017. For the data collection, the researchers contacted the administrators of nurseries, explained the purpose and importance of the study to the administrators of each nursery, and got permission to conduct data collection within the nursery. The administrators of each nursery contacted mothers who met inclusion criteria and received consent to provide their contact information to the research team. After obtaining the contact list, the researchers contacted and explained the purpose of the study to each participant, and received their signed informed consent. It took approximately 20 min for each participant to complete the questionnaires. Afterwards, each participant was given a gift card of appreciation for their participation.

Prior to data collection, ethical approval was received from the institutional review board (IRB) of A university medical center in Korea (IRB No. *****-MED-SUR-17–233). Participants were given a detailed explanation of the purpose, process, rewards for participation, guarantees for anonymity, and voluntary participation. Written informed consent was obtained from all participants.

### Data analysis

Data were analyzed using SPSS version 23.0. The normality of the study variables were examined using the Kolmogorov–Smirnov test, with *p* > 0.05 and indicated that the data were normally distributed. Descriptive statistics were used to define the participants' demographic characteristics, parenting efficacy, childcare support from husbands, parenting stress, social support, role conflict between parenting and work, and family friendly workplace systems and use of childbirth and parenting friendly workplace systems. Independent samples t‐test, analysis of variance, and Scheffe test were conducted to identify differences in participants' parenting efficacy according to general characteristics. Pearson's correlation coefficients were calculated to identify relationships between parenting efficacy and other study variables. Lastly, in order to examine the factors affecting parenting efficacy, a hierarchical multiple regression analysis was conducted. A significance level of p < 0.05 was used for data analysis.

## Results

### Participant characteristics and PSE according to general characteristics

The majority of mothers were 30–39 years old (82.9%, n = 247), and the mean age of the last child was 17.12 (± 9.45) months. More than half were primipara (59.1%, n = 176) and have graduated from university (57.7%, n = 172). Most mothers lived with their spouse only (87.2%, n = 260). Mothers who rated themselves as primary caregivers (63.4%, n = 189) were more than those who rated others as primary caregiver (36.6%, n = 109). Most of the occupations were profession (45.0%, n = 134) and office worker (35.9%, n = 107), and more than half of the mothers were in stable employment (64.1%, n = 191). The most common duration of employment was less than 5 years (41.9%, n = 124), followed by 5 ~ 10 years (37.8%, n = 112).

Regarding differences of PSE according to general characteristics, PSE was higher in working mothers who rated themselves as the primary caregiver than those who did not rate themselves as the primary caregiver (t = 2.16, *p* = 0.032). Working mothers with less than 5 years of employment had higher PSE than those with more than 5 years of employment (F = 3.82, *p* = 0.023) (Table 1).

**Table 1:** Independent t-test and one way ANOVA of the parenting self-efficacy according to general characteristics (N = 298)

Characteristics	Categories	n (%)	Parenting self-efficacy	
			M ± SD	t or F (*p*)
Age of mother (yrs)	20–29	25 (8.4)	31.76 ± 5.07	0.22 (.800)
	30–39	247 (82.9)	31.85 ± 4.03	
	40–49	26 (8.7)	32.38 ± 2.68	
Parity	Primipara	176 (59.1)	31.69 ± 3.82	− 0.99 (.321)
	Multipara	122 (40.9)	32.16 ± 4.29	
Age of last child	1–12	109 (36.6)	32.01 ± 4.35	0.08 (.920)
(months)	13–24	120 (40.3)	31.83 ± 3.99	
(M ± SD, 17.12 ± 9.45)	25–36	69 (23.2)	31.78 ± 3.55	
Family type	Nuclear	260 (87.2)	31.83 ± 3.86	− 0.58 (.565)
	Extended	38 (12.8)	32.24 ± 5.04	
Primary caregiver	Herself	189 (63.4)	32.26 ± 3.86	2.16 (.032)
	Others	109 (36.6)	31.23 ± 4.22	
Household income^*^	< 400	61 (20.5)	31.20 ± 4.02	1.81 (.166)
(10,000won/month)	400–600	131 (44.1)	32.34 ± 4.35	
	≥ 600	105 (35.4)	31.72 ± 3.54	
Education	High school	28 (9.4)	32.39 ± 3.55	0.26 (.853)
	College	53 (17.8)	31.63 ± 4.20	
	University	172 (57.7)	31.84 ± 4.07	
	Graduate school	45 (15.1)	32.07 ± 3.97	
Occupation	Self-employment	13 (4.4)	32.85 ± 3.13	1.85 (.120)
	Service/Sales	29 (9.7)	32.41 ± 4.65	
	Office worker	107 (35.9)	31.45 ± 3.99	
	Profession	134 (45.0)	31.78 ± 4.02	
	Others	15 (5.0)	34.13 ± 2.83	
Duration of employment	< 5	124 (41.9)	32.60 ± 4.09^a^	3.82 (.023)
(yrs)	5–10	112 (37.8)	31.49 ± 3.84^b^	a > b
	≥ 10	60 (20.3)	31.07 ± 4.05^b^	
Stability of employment	Yes	191 (64.1)	31.84 ± 3.99	− 0.25 (.806)
	No	107 (35.9)	31.96 ± 4.10	

^*^Valid percent

### Levels of PSE and affecting variables

The mean score (standard deviation (SD)) of PSE was 31.89 (4.02). Also, in the individual level, mean scores (SD) of perceived health status, maternal role satisfaction, and parenting stress were 5.84 (2.20), 6.0 (1.74), and 97.93 (21.46), respectively. In the micro-system level, the average of mean scores (SD) of work-parent role conflict, parenting support by spouse, and social support were 24.03 (6.68), 52.31 (9.09), and 58.06 (9.26), respectively. In the exo-system level, mean scores (SD) of childbirth & parenting friendly workplace system were 2.13 (1.75) (Table 2).

**Table 2:** Descriptive statistic of the study variables (N = 298)

Categories	Study variables	Range	Min	Max	M ± SD
Dependent variable	Parenting self-efficacy	9–45	20	45	31.89 ± 4.02
Individual level	Perceived health status	1–10	0	10	5.84 ± 2.20
	Maternal role satisfaction	1–10	0	10	6.00 ± 1.74
	Parenting stress	32–160	32	156	97.93 ± 21.46
Micro-system level	Work-parent role conflict	8–40	8	40	24.03 ± 6.68
	Parenting support by spouse	14–70	23	70	52.31 ± 9.09
	Social support	19–76	33	76	58.06 ± 9.26
Exo-system level	Childbirth and parenting friendly workplace system	0–7	0	7	2.13 ± 1.75

### Relationships between PSF and affecting variables

PSE had positive relationships with perceived health status (r = 0.19, *p* = 0.001), maternal role satisfaction (r = 0.40, *p* < 0.001), parenting support by spouse (r = 0.29, *p* < 0.001), and social support (r = 0.26, *p* < 0.001), while it had a negative relationship with parenting stress (r = − 0.22, *p* < 0.001), work-parent role conflict (r = − 0.26, *p* < 0.001) and childbirth and parenting friendly workplace systems (r = − 0.14, *p* = 0.019) (Table 3).

**Table 3:** Correlation analysis between study variables (N = 298)

	Parenting self-efficacy r (*p*)	Perceived health Status r (*p*)	Maternal role satisfaction r (*p*)	Parenting stress r (*p*)	Work-parent role conflict (*p*)	Parenting support by spouse r (*p*)	Social support r (*p*)	Workplace system r (*p*)
Parenting self-efficacy	1							
Perceived health status	.19 (.001)	1						
Maternal role satisfaction	.40 (< .001)	.40 (< .001)	1					
Parenting Stress	− .22 (< .001)	− .41 (< .001)	− .36 (< .001)	1				
Work-parent role conflict	− .26 (< .001)	− .22 (< .001)	− .34 (< .001)	.55 (< .001)	1			
Parenting supportby spouse	.29 (< .001)	.19 (.001)	.27 (< .001)	− .35 (< .001)	− .30 (< .001)	1		
Social support	.26 (< .001)	.30 (< .001)	.31 (< .001)	− .46 (< .001)	− .36 (< .001)	.45 (< .001)	1	
Workplace system	− .14 (.019)	.01 (.870)	− .03 (.575)	− .09 (.107)	.05 (.431)	.08 (.155)	.12 (.048)	1

Workplace system: childbirth and parenting friendly workplace system

### Factors influencing PSE of working mothers

In model 1, including only general characteristics, PSE of working mothers with more than 10 years of employment was lower than that of working mothers with less than 5 years of employment (β = − 0.15, *p* = 0.019).

In model 2, including the individual level and general characteristics, PSE of working mothers was influenced by maternal role satisfaction (β = 0.33, *p* < 0.001) and parenting stress (β = − 0.12, *p* = 0.045) in the individual level. Also PSE of working mothers with more than 5 years and less than 10 years of employment (β = − 0.14, *p* = 0.016) as well as working mothers with more than 10 years of employment (β = − 0.14, *p* = 0.022) was lower than that of working mothers with less than 5 years of employment. On the other hand, working mothers who rated themselves as the primary caregiver had higher PSE scores compared to those who did not rate themselves as the primary caregiver (β = 0.12, *p* = 0.035).

In model 3, which included the individual level, micro-system level, and general characteristics, PSE of working mothers was influenced by maternal role satisfaction (β = 0.28, *p* < 0.001) in the individual level and parenting support by spouse (β = 0.18, *p* = 0.003) in the micro-system level. As in model 2, PSE of working mothers with more than 5 years and less than 10 years of employment (β = − 0.15, *p* = 0.011) and working mothers with more than 10 years of employment (β = − 0.14, *p* = 0.017) was lower than that of working mothers with less than 5 years of employment. Working mothers who rated themselves as the primary caregiver had higher PSE compared to those who did not rate themselves as the primary caregiver (β = 0.12, *p* = 0.035).

In model 4, including general characteristics and all ecological factors, PSE of working mothers was influenced by maternal role satisfaction (β = 0.27, *p* < 0.001) in the individual level and parenting support by spouse (β = 0.19, *p* = 0.002) in the micro-system level. Also working mothers who rated themselves as the primary caregiver had higher PSE scores compared to those who did not rate themselves as the primary caregiver (β = 0.13, *p* = 0.022). The explanatory power of this predictive model was 23.0% (Table 4).

**Table 4:** Hierarchical multiple regression of the factors influencing parenting self-efficacy of working mothers (N = 298)

Categories	Variables	Model 1				Model 2				Model 3				Model 4			
		B	SD	β	t (*p*)	B	SD	β	t (*p*)	B	SD	β	t (*p*)	B	SD	β	t (*p*)
Constant		31.96	0.50		63.50 (< .001)	29.23	1.80		16.26 (< .001)	22.78	2.76		8.27 (< .001)	22.97	2.75		8.37 (< .001)
General characteristics	Primary caregiver—herself^*^	0.90	0.50	.11	1.80 (.072)	1.00	0.47	.12	2.12 (.035)	1.04	0.47	.12	2.21 (.028)	1.08	0.47	.13	2.31 (.022)
	Duration of employment 5–10^§^	− 1.04	0.54	–.12	− 1.94 (.053)	− 1.19	0.49	–.14	− 2.42 (.016)	− 1.24	0.48	–.15	− 2.57 (.011)	− 0.96	0.51	–.11	− 1.88 (.061)
	Duration of employment ≥ 10^§^	− 1.54	0.65	–.15	− 2.36 (.019)	− 1.37	0.60	–.14	− 2.30 (.022)	− 1.40	0.59	–.14	− 2.39 (.017)	− 1.09	0.61	–.11	− 1.80 (.074)
Individual level	Perceived health status					0.08	0.11	.04	0.71 (.481)	0.07	0.11	.04	0.64 (.520)	0.07	0.11	.04	0.59 (.554)
	Maternal role satisfaction					0.76	0.14	.33	5.36 (< .001)	0.65	0.14	.28	4.56 (< .001)	0.63	0.14	.27	4.45 (< .001)
	Parenting stress					− 0.02	0.01	–.12	− 2.02 (.045)	− 0.01	0.01	–.02	− 0.27 (.786)	− 0.01	0.01	–.03	− 0.45 (.650)
Micro-system level	Work-parent role conflict									− 0.03	0.04	–.05	− 0.80 (.423)	− 0.24	0.04	–.04	− 0.61 (.541)
	Parenting support by spouse									0.08	0.03	.18	3.02 (.003)	0.08	0.03	.19	3.09 (.002)
	Social support									0.03	0.03	.07	1.10 (.285)	0.04	0.03	.08	1.23 (.220)
Exo-system level	Workplace system													− 0.24	0.13	–.10	− 1.79 (.074)
R		.196				.454				.499				.507			
R^2^		.038				.206				.249				.258			
Adjusted R^2^		.028				.189				.224				.230			
F (*p*)		3.73 (.012)				12.02 (< .001)				10.12 (< .001)				9.50 (< .001)			

Workplace system: childbirth and parenting friendly workplace system. *Reference group: primary caregiver-others; ^§^ reference group: duration of employment < 5

## Discussion

This study was conducted to identify the factors influencing PSE of working mothers from the individual level, micro-system level, and exo-system level based on the ecological model.

The results of this study showed that in the individual level, the higher maternal role satisfaction, the higher PSE of working mothers. This result was consistent with previous studies which reported that maternal role satisfaction was associated with maternal PSE [40, 41]. During our literature search, we could not find any studies that reported results contradicting the result of this study. Maternal role satisfaction means a kind of satisfaction and pleasure that a woman experiences in interacting with her infant and in carrying out the maternal role [42]. Maternal role satisfaction is closely related with maternal PSE [41], maternal PSE is difficult to achieve if a mother is not satisfied with her maternal role [40]. Therefore, it is necessary to increase maternal role satisfaction to increase the maternal PSE of working mothers.

Maternal role satisfaction can have a considerable impact on the quality of parenting behaviors [43]; mothers with higher level of satisfaction in their parental role practiced more positive parenting behaviors [44]. Mothers who are satisfied with their maternal role were less likely to report anxiety [45] and psychological distress [46]. Based on these findings and the results of this study, it appears that an increase in maternal role satisfaction increases the maternal PSE of working mothers as well as the well-being of working mothers and their children.

Lack of knowledge and awareness about the maternal role is the most important factor in rejecting the maternal role and reduces maternal role satisfaction, so it is necessary to educate and support mothers about maternal roles [47]. Previous studies reported that mothers educated about maternal role increased maternal identity [48] and maternal role satisfaction [47]. Therefore, to increase PSE of working mothers, it is necessary to provide educational interventions about maternal roles for increasing the awareness and satisfaction of the maternal role.

The results of this study showed that in the micro-system level, the higher parenting support by spouse, the higher PSE of working mothers. This result is consistent with previous studies which reported that maternal PSE was significantly influenced by parenting support from spouse [13, 49]. During our literature search, we could not find any studies that reported results contradicting the result of this study. Social support is an important factor increasing maternal PSE [6, 50] and support from a spouse is the main source of social support for mothers [6, 51]. Working mothers have to balance work and child care and are also often responsible for household chores, so their support needs are greater than those of not-working mothers. Recently, because family structures have changed to favor nuclear families in Korea, spousal support is now the primary parenting support source that working mothers receive within the family [24].

However, many mothers perceived that support from their spouse was insufficient [16, 41, 52]. In traditional Asian families, fathers tend to place greater emphasis on their work and fathers' parenting support was primarily limited to fiscal support [11, 53]. But, as the rate of working mothers increase and social expectations about the roles of father in the family change, the need for fathers’ participation in child care has increased. Mothers' perception about fathers' participation in parenting may contribute significantly to reducing their parenting burden [16]. Therefore, it is necessary to provide various strategies for fathers' active participation in parenting and to increase awareness of the importance of fathers' participation in childrearing. In additions, fathers who want to participate in parenting often do not have enough education or experience in parenting to provide substantial help [54]. This is further exacerbated by the fact that most intervention programs on parenting are focused on mothers and often do not include fathers at all [48]. Fathers' participation in parenting programs needs to be encouraged to improve their knowledge and skills on how to support mothers and how to improve their PSE [41]. Therefore, it is necessary to include fathers as well as mothers in intervention programs for parenting and to activate online or mobile education programs so that they can obtain the necessary information regardless of time or place.

The results of this study showed that working mothers who rated themselves as the primary caregiver had higher PSE compared to those who did not rate themselves as the primary caregiver. We could not find any previous studies that applied the same concept as this study in identifying the influencing factors of maternal PSE, therefore it is difficult to directly compare the results of this study with previous studies. The number of children was associated with higher PSE [55] and multiparas showed higher PSE than primiparas [12, 15]. In this study, although there was no statistical significance, multipara showed higher PSE than primipara. These results are consistent with our understanding that parenting-related experiences increased PSE and are also consistent with Bandura's theory that mastery experiences enhance self-efficacy [6, 55]. Mothers, as a primary caregiver, feel responsibility for maintaining and promoting their children's well-being and development and providing sufficient stimulation and cultural opportunities to learn and grow into successful individuals [56]. Working mothers who are the primary caregiver also feel this responsibility and participate in various childcare activities. However, even though working mothers who are not primary caregivers recognize this responsibility, they are not primary caregivers, so their participation in parenting may be less than that of working mothers who are primary caregivers. Therefore, it can be said that working mothers who are primary caregivers have more experience in parenting than working mothers who are not primary caregivers, and this results in higher PSE of working mothers who are primary caregivers.

However, mothers who are the primary caregiver may also be at greater risk of experiencing a high level of parenting stress [57], and the accumulation of parental stress may contribute to the development of maternal burnout syndrome [58]. Working mothers who are the primary caregiver have to combine work and childcare, so the risk of parenting stress and the resulting maternal burnout syndrome is likely greater. Therefore, it is necessary to reduce the responsibilities or burden of parenting of working mothers who are primary caregivers. In recent years, co-parenting has been encouraged as a way to reduce the responsibilities or burden of parenting [59]. Co-parenting refers to the ways that parents work together in their roles as parents and is characterized by how partners cooperate and support each other, rather than by undermining one another's efforts in parenting activities, responsibilities, and roles as parents [60]. Co-parenting is associated with positive parent and child outcomes, like as enhancing PSE and promoting parent–child interactions [61]. Therefore, it is necessary to encourage co-parenting for working mothers who are the primary caregiver and their spouses, and to provide various intervention programs to support co-parenting amongst this group.

## Limitations

This study was conducted by convenience sampling and used a self-report instrument within a cross-sectional design, thus there is a limitation in our ability to explain a causal link to predict PSE and to control the effects of confounding variables or to rule out errors of sampling. Therefore, further research is needed using random sampling methods. Also, although there are valid and reliable multi-question instruments, the one item measurement for perceived health status and maternal role satisfaction were used to avoid response burden to measure many variables of ecological model. Despite this limitation, this work is meaningful to explore the PSE of working mothers and various affecting factors of it based on the ecological model. Also, this work contributes to improve understanding about the phenomenon of maternal adaptation of working mothers, and gives ideas for intervention studies to support the successful adaptation to motherhood.

## Conclusion

In this study, the higher maternal role satisfaction (at the individual levels) and the higher parenting support by spouse (in the micro-system level), the higher parenting self-efficacy of working mothers. In addition, working mothers who were the primary caregiver had higher parenting self-efficacy compared to those who were not the primary caregiver. Therefore, educational interventions aimed at increasing the awareness and satisfaction of maternal role and various strategies to support fathers' active participation in parenting should be provided. In addition, practical interventions such as co-parenting that can reduce the overall burden of parenting while increasing parenting self-efficacy of working mothers who are the primary caregiver should also be implemented.

## Data Availability

The datasets used and analyzed during the current study are available from the corresponding author on reasonable request.
